# The Burden of Progressive Supranuclear Palsy on Patients, Caregivers, and Healthcare Systems by PSP Phenotype: A Cross-Sectional Study

**DOI:** 10.3389/fneur.2022.821570

**Published:** 2022-07-04

**Authors:** Demetris Pillas, Alexander Klein, Teresa Gasalla, Andreja Avbersek, Alexander Thompson, Jack Wright, Jennifer Mellor, Anna Scowcroft

**Affiliations:** ^1^UCB Pharma, Brussels, Belgium; ^2^Rare Disease Franchise, Adelphi Real World, Bollington, United Kingdom

**Keywords:** progressive supranuclear palsy, PSP, PSP phenotype, disease burden, mortality

## Abstract

Progressive supranuclear palsy (PSP) is a rare, relentlessly progressive, ultimately fatal neurodegenerative brain disease. The objective of this study was to assess the burden of PSP on patients, caregivers, and healthcare systems by PSP phenotype. Data were drawn from the Adelphi PSP Disease Specific Programme™, a cross-sectional study of neurologists and people living with PSP in the United States of America, France, Germany, Italy, Spain, and the United Kingdom. All people living with PSP with a reported phenotype were included. PSP phenotype was reported for 242 patients (mean age: 70.2 years, 58% male): PSP-Richardson's syndrome, *n* = 96; PSP-predominant Parkinsonism, *n* = 88; PSP-predominant corticobasal syndrome, *n* = 28; PSP-predominant speech/language disorder, *n* = 12; PSP-progressive gait freezing, *n* = 9; PSP-predominant frontal presentation, *n* = 9. Most patients reported impaired cognitive, motor, behavioral and ocular functionality; 67–100% of patients (across phenotypes) had moderate-to-severe disease at the time of data collection. Post-diagnosis, the majority were provided with a visual and/or mobility aid (55–100%, across phenotypes), and/or required home modification to facilitate their needs (55–78%, across phenotypes). Patients required multiple types of healthcare professionals for disease management (mean 3.6–4.4, across phenotypes), and the majority reported receiving care from at least one caregiver (mean 1.3–1.8, across phenotypes). There is a high burden on patients, caregivers, and healthcare systems across all PSP phenotypes. Although phenotypes manifest different symptoms and are associated with different diagnostic pathways, once diagnosed with PSP, patients typically receive similar care.

## Introduction

Progressive supranuclear palsy (PSP) is a rare, relentlessly progressive, ultimately fatal neurodegenerative brain disease (1–3). PSP was originally described in its most common clinical form, now termed PSP-Richardson's syndrome (PSP-RS) (4). Since then, further clinical phenotypes have been described to cover the observed heterogeneity in clinical characteristics of people living with PSP (5), particularly in the early stages of the disease, culminating in the definition of eight PSP phenotypic variants according to the Movement Disorder Society (MDS) Criteria (6): PSP-RS, PSP with predominant Parkinsonism (PSP-P), PSP with predominant corticobasal syndrome (PSP-CBS), PSP with predominant speech/language disorder (PSP-SL), PSP with progressive gait freezing (PSP-PGF), PSP with predominant frontal presentation (PSP-F), PSP with predominant ocular motor dysfunction (PSP-OM) and PSP with predominant postural instability (PSP-PI). It has been posited that most of the PSP phenotypic variants progress to develop some or all of the typical clinical features of PSP-RS (7).

A substantial evidence base now exists describing the overall burden of PSP (8); however, this evidence base has been largely limited to people living with PSP-RS (8) and PSP-P (9). This is unsurprising considering that PSP-RS and PSP-P are the most commonly presenting phenotypic variants of PSP (>50% and 14–35% of cases, respectively) (10–12). The scarcity of studies on the remaining variants renders any systematic comparisons of disease burden by various phenotypic variants difficult. To address this evidence gap, we used a large study of people living with PSP whose PSP phenotype was recorded by neurologists, with the aim of assessing the disease burden on patients, caregivers, and healthcare systems by PSP phenotype.

## Materials and Methods

### Study Design

The study, the Adelphi PSP Disease Specific Programme (DSP™), was carried out in 2018 in the United States of America (USA) and EU5 [France, Germany, Italy, Spain, and the United Kingdom (UK)]. DSPs™ are large, multinational surveys conducted in clinical practice that describe current disease management, disease-burden impact, and associated treatment effects (clinical and physician-perceived). The DSP™ is a point-in-time survey of physicians and their patients presenting in a real-world clinical setting (13). There were no follow-up procedures in this DSP™. More details relating to the methodology of this particular DSP™ have been reported elsewhere (14).

### Data Collection

Between July and November 2018, neurologists and movement disorder specialists were invited to complete record forms for their next eligible patients (the first patients post-study initiation) to visit them for routine care. The main component of data collection in this study was the Patient Record Forms (PRFs), which were detailed records completed by the physician for their patients presenting with PSP. Each PRF took ~20–25 min to complete, and contained detailed questions on patient demographic and clinical characteristics, key parameters related to the patient journey (such as symptom presentation, disease severity, PSP phenotype), and parameters relating to patient management and healthcare resource utilization [healthcare professional (HCP) and caregiver interactions, provision of medical devices].

Completion of the physician-reported questionnaire was undertaken through consultation of existing patient clinical records, as well as the judgement and diagnostic skills of the respondent neurologist/movement disorder specialist, which is entirely consistent with decisions made in routine clinical practice. The survey was designed to facilitate understanding of real-world clinical practice, and thus physicians could only report on data available at the time of the consultation. No tests, treatments, or investigations were performed as part of this survey. Physician participation was financially incentivized according to fair market research rates.

### Identification of Cases

Neurologists and movement disorder specialists were eligible to participate if: they were personally responsible for treatment decisions and management of people living with PSP, their primary specialty was neurology (inclusive of movement disorder specialists), and they were currently managing at least 1 person living with PSP. In the overall DSP™, patients were eligible for inclusion if they were aged ≥18 years, had a physician-confirmed diagnosis of PSP, and visited the recruited neurologist/movement disorder specialist.

Overall, the DSP™ recruited 203 neurologists/movement disorder specialists who provided data on 892 people living with PSP. From this overall patient sample, this study focused on the subset of people living with a PSP phenotype recorded by their neurologist/movement disorder specialist.

In order to achieve geographic representation/data generalizability, the study sought to recruit physicians from a range of regions/centers.

### Data on Disease Burden

From the wider data collection conducted in this DSP™, this study used solely physician-reported data covering disease burden on people living with PSP, their caregivers and on the wider healthcare system since diagnosis. Patient burden was assessed *via* disease severity (physician-reported on a 3-point scale: mild, moderate, severe), impairment of functionality in the motor, ocular, cognitive, and behavioral domains (physician-reported across functional domains on a 5-point scale: completely functional, functional, neither, impaired, completely impaired), and by PSP-related symptom presentation (number of symptoms, most commonly presenting symptoms), as reported by the participating physician. Caregiver burden was assessed *via* the presence of a caregiver and mean number of caregivers, whether a professional caregiver was provided, the total hours of care required per week, and the most common types of assistance required on behalf of the caregiver, as reported by the participating physician or caregiver. It is important to note that, at enrolment, all physicians were instructed to complete the study questionnaires on the day the patient (and accompanying caregiver when applicable) was consulted; therefore, all parties (physician, patient, caregiver) were engaged in the process of data recording / completion. Burden to the healthcare system was reported by participating physicians using the patients' hospitalization history since diagnosis, the number and specializations of HCPs involved in patient management, total consultations provided, whether patient aids relating to PSP were provided (mobility and/or visual aids), and whether a home modification was performed to facilitate patient needs.

### Statistical Analysis

Inferential statistics aiming to compare differences between phenotypic variants were not performed due to the small sample sizes of the non-PSP-RS and PSP-P variants. All variables were analyzed descriptively using Stata statistical software version 16.1 (StataCorp. 2019. Stata Statistical Software: Release 16. College Station, TX: StataCorp LLC). Means and standard deviations were reported for continuous variables, and frequency counts and percentages for categorical variables. Only the most common comorbidities were reported (occurring in >10% of the overall sample of patients with PSP). No missing data were observed for the majority of variables. In the few variables where minimal missing data were observed, meaning that the base of patients for analysis could vary, this was reported separately for each variable.

### Ethics Considerations

The survey was performed in full accordance with relevant legislation at the time of data collection, including the US Health Insurance Portability and Accountability Act 1996 (15) and Health Information Technology for Economic and Clinical Health Act legislation (16). Data collection was undertaken in line with European Pharmaceutical Marketing Research Association guidelines (17). Using a check box, patients provided informed consent for use of their anonymized and aggregated data for research and publication in scientific journals. Data were collected so that patients and physicians could not be identified directly; all data were aggregated and de-identified. This research obtained ethics approval from the Salus Institutional Review Board, study protocol number AG 8445.

## Results

### Clinical and Demographic Characteristics

A total of 242 people living with PSP (27% of the 892 patients on which data were collected) met the overall DSP™ inclusion criteria and also had their PSP phenotype recorded. These data were derived from 83 neurologists/movement disorder specialists (41% of the 203 in total recruited) who assigned a predominant phenotype to patients.

From the spectrum of phenotypes reported in the MDS Criteria© (6), six (out of eight defined) were reported in this data, with the following distribution: PSP-RS: 96 (40%), PSP-P: 88 (36%), PSP-CBS: 28 (12%), PSP-SL: 12 (5%), PSP-PGF: 9 (4%), and PSP-F: 9 (4%). No patients were reported with PSP-OM or PSP-PI. At the time of the physician survey, time since diagnosis varied across patients, with the median ranging from 8.0 to 22.5 months, according to phenotype.

The mean age of patients ranged from 66.2 to 73.7 years across PSP phenotypes. Patients were primarily male (58%) and of White/Caucasian ethnic background (Table 1). One third to a half of patients were overweight or obese, a characteristic observed across all phenotypes. A similar comorbidity profile was observed across phenotypes, with seven comorbid conditions reported in >10% of the overall patient sample.

**Table 1:** Patient demographics and clinical characteristics, by PSP phenotype.

	**PSP-RS**	**PSP-P**	**PSP-CBS**	**PSP-SL**	**PSP-PGF**	**PSP-F**	**Total**
	**(*n* = 96)**	**(*n* = 88)**	**(*n* = 28)**	**(*n* = 12)**	**(*n* = 9)**	**(*n* = 9)**	**(*n* = 242)**
**Age, years**							
Mean (SD)	70.4 (7.5)	69.9 (9.6)	69.5 (9.0)	66.2 (11.0)	73.7 (4.4)	73.7 (8.1)	70.2 (8.6)
**Gender**, ***n*** **(%)**							
Male	56 (58%)	56 (64%)	12 (43%)	8 (67%)	4 (44%)	4 (44%)	140 (58%)
**Country**, ***n*** **(%)**							
France	13 (14%)	8 (9%)	4 (14%)	1 (8%)	4 (44%)	0 (0%)	30 (12%)
Germany	25 (26%)	17 (19%)	3 (11%)	1 (8%)	1 (11%)	1 (11%)	48 (20%)
Italy	21 (22%)	13 (15%)	5 (18%)	4 (33%)	2 (22%)	3 (33%)	48 (20%)
Spain	12 (12%)	18 (20%)	5 (18%)	0 (0%)	0 (0%)	1 (11%)	36 (15%)
UK	9 (9%)	17 (19%)	8 (29%)	4 (33%)	2 (22%)	3 (33%)	43 (18%)
US	16 (17%)	15 (17%)	3 (11%)	2 (17%)	0 (0%)	1 (11%)	37 (15%)
**Ethnicity**, ***n*** **(%)**							
White/Caucasian	91 (95%)	74 (84%)	26 (93%)	11 (92%)	8 (89%)	9 (100%)	219 (90%)
**Time since diagnosis, months**
At survey, median (IQR)	22.0 (23.8)	18.0 (22.0)	18.0 (27.0)	8.0 (8.0)	11.0 (19.0)	22.5 (29.3)	19.0 (22.8)
Missing	2 (2%)	1 (1%)	1 (4%)	1 (8%)	0 (0%)	1 (11%)	6 (2%)
**BMI ≥25 kg/m^2^, *n* (%)**							
Overweight/obese	38 (40%)	45 (51%)	9 (32%)	7 (58%)	4 (44%)	3 (33%)	106 (44%)
**Number of comorbidities**							
Mean (SD)	1.6 (1.3)	2.0 (1.3)	1.9 (1.4)	1.8 (1.2)	1.8 (1.0)	3.0 (2.6)	1.8 (1.4)
Common comorbidities^*^, ***n*** **(%)**							
Depression	27 (28%)	26 (30%)	9 (32%)	2 (17%)	2 (22%)	2 (22%)	68 (28%)
Diabetes	17 (18%)	23 (26%)	10 (36%)	0 (0%)	1 (11%)	3 (33%)	54 (22%)
Anxiety	16 (17%)	15 (17%)	3 (11%)	3 (25%)	2 (22%)	1 (11%)	40 (17%)
Cerebrovascular disease	13 (14%)	17 (19%)	0 (0%)	3 (25%)	1 (11%)	4 (44%)	38 (16%)
Dementia	8 (8%)	11 (13%)	6 (21%)	1 (8%)	0 (0%)	3 (33%)	29 (12%)
Peripheral vascular disease	10 (10%)	12 (14%)	4 (14%)	1 (8%)	1 (11%)	1 (11%)	29 (12%)
Myocardial infarction	13 (14%)	5 (6%)	3 (11%)	1 (8%)	0 (0%)	3 (33%)	25 (10%)

* *Comorbidities reported in >10% of all patients. IQR, interquartile range; PSP, progressive supranuclear palsy; PSP-CBS, PSP-predominant corticobasal syndrome; PSP-F, PSP-predominant frontal presentation; PSP-P, PSP-predominant Parkinsonism; PSP-PGF, PSP-progressive gait freezing; PSP-RS, PSP-Richardson's syndrome; PSP-SL, PSP-predominant speech/language disorder; SD, standard deviation; UK, United Kingdom; US, United States*.

### Patient Burden

The majority of patients across all PSP phenotypes had moderate-to-severe disease at the time of data collection (67–100% of patients, across phenotypes; Table 2). For all functional domains evaluated (motor, ocular, cognitive, behavioral), at least 45% of patients were reported with impaired functionality, with few differences observed between phenotypes. Impaired motor and ocular functionality were most commonly reported (76 and 67% of patients, respectively).

**Table 2:** Patient burden (disease severity, functional impairment, and symptom presentation), by PSP phenotype.

	**PSP-RS**	**PSP-P**	**PSP-CBS**	**PSP-SL**	**PSP-PGF**	**PSP-F**	**Total**
	**(*n* = 96)**	**(*n* = 88)**	**(*n* = 28)**	**(*n* = 12)**	**(*n* = 9)**	**(*n* = 9)**	**(*n* = 242)**
**Disease severity**, ***n*** **(%)**							
Mild	21 (22%)	18 (20%)	2 (7%)	4 (33%)	1 (11%)	0 (0%)	46 (19%)
Moderate	34 (35%)	51 (58%)	13 (46%)	5 (42%)	5 (56%)	4 (44%)	112 (46%)
Severe	41 (43%)	19 (22%)	13 (46%)	3 (25%)	3 (33%)	5 (56%)	84 (35%)
**Impaired functionality**, ***n*** **(%)**							
Motor	75 (78%)	65 (74%)	22 (79%)	4 (33%)	9 (100%)	9 (100%)	184 (76%)
Ocular	72 (75%)	56 (64%)	16 (57%)	4 (33%)	6 (67%)	7 (78%)	161 (67%)
Cognitive	46 (48%)	49 (56%)	17 (61%)	7 (58%)	3 (33%)	9 (100%)	133 (55%)
Behavioral	42 (44%)	38 (43%)	12 (43%)	6 (50%)	3 (33%)	9 (100%)	110 (45%)
**Number of PSP-related symptoms**							
Mean (SD)	21.0 (9.8)	18.2 (9.5)	21.0 (11.6)	15.5 (9.7)	14.8 (7.8)	29.4 (11.3)	19.8 (10.2)
Common symptoms^*^, ***n*** **(%)**							
Difficulty walking	92 (96%)	82 (93%)	25 (89%)	9 (75%)	9 (100%)	9 (100%)	226 (93%)
Blepharospasm	81 (84%)	77 (88%)	24 (86%)	8 (67%)	9 (100%)	9 (100%)	208 (86%)
Rigidity	75 (78%)	67 (76%)	25 (89%)	7 (58%)	8 (89%)	8 (89%)	190 (79%)
Dysphagia	75 (78%)	67 (76%)	21 (75%)	10 (83%)	8 (89%)	9 (100%)	190 (79%)
Difficulty looking up	75 (78%)	65 (74%)	23 (82%)	7 (58%)	5 (56%)	8 (89%)	183 (76%)
Difficulty looking down	78 (81%)	60 (68%)	20 (71%)	7 (58%)	6 (67%)	8 (89%)	179 (74%)
Slow thinking/reasoning	71 (74%)	64 (73%)	22 (79%)	10 (83%)	3 (33%)	9 (100%)	179 (74%)
Insomnia	67 (70%)	69 (78%)	19 (68%)	7 (58%)	4 (44%)	8 (89%)	174 (72%)
Incontinence	70 (73%)	60 (68%)	18 (64%)	5 (42%)	6 (67%)	9 (100%)	168 (69%)
Irritability	54 (56%)	59 (67%)	19 (68%)	6 (50%)	4 (44%)	7 (78%)	149 (62%)
Confusion/disorientation	56 (58%)	52 (59%)	17 (61%)	7 (58%)	2 (22%)	8 (89%)	142 (59%)
Reduced blinking	65 (68%)	44 (50%)	16 (57%)	5 (42%)	6 (67%)	6 (67%)	142 (59%)
Muscle twitches/spasms	57 (59%)	50 (57%)	21 (75%)	3 (25%)	3 (33%)	3 (33%)	137 (57%)
Loss of balance/falling	66 (69%)	33 (38%)	15 (54%)	2 (17%)	6 (67%)	5 (56%)	127 (52%)

* *Symptoms reported in >50% of all patients. PSP, progressive supranuclear palsy; PSP-CBS, PSP-predominant corticobasal syndrome; PSP-F, PSP-predominant frontal presentation; PSP-P, PSP-predominant Parkinsonism; PSP-PGF, PSP-progressive gait freezing; PSP-RS, PSP-Richardson's syndrome; PSP-SL, PSP-predominant speech/language disorder; SD, standard deviation*.

For all PSP phenotypes, the majority of patients reported multiple symptoms covering a wide range of disability since diagnosis. A total of 14 different symptom types were reported in >50% of all patients. Specific symptoms were reported in almost all patients, across all phenotypes. Overall, difficulty walking, blepharospasm/involuntary blinking, rigidity, and dysphagia were the most commonly reported symptoms.

### Caregiver Burden

At the time the survey was taken, almost 3 in 4 patients were reported as having a caregiver (range: 62–100%, across phenotypes), with similar results reported across phenotypes in respect to the number of caregivers providing care (1.3–1.8, across phenotypes; Table 3). Similar results across phenotypes were observed in terms of the proportion of patients who required a professional caregiver (22–36%) at the time the survey was taken, with the exception of people living with PSP-F, for whom 75% required a professional caregiver.

**Table 3:** Caregiver burden, by PSP phenotype.

	**PSP-RS**	**PSP-P**	**PSP-CBS**	**PSP-SL**	**PSP-PGF**	**PSP-F**	**Total^†^**
	**(*n* = 96)**	**(*n* = 88)**	**(*n* = 28)**	**(*n* = 12)**	**(*n* = 9)**	**(*n* = 9)**	**(*n* = 242)**
**Presence of a caregiver**, ***n*** **(%)**							
Patient has caregiver	75 (79%)	53 (62%)	23 (85%)	9 (75%)	6 (67%)	8 (100%)	174 (73%)
Missing	1 (1%)	2 (2%)	1 (4%)	0 (0%)	0 (0%)	1 (11%)	5 (2%)
**Number of caregivers**							
Mean (SD)	1.7 (0.7)	1.5 (0.7)	1.5 (0.6)	1.3 (0.7)	1.7 (0.8)	1.8 (1.0)	1.6 (0.7)
Missing	21 (22%)	35 (40%)	5 (18%)	3 (25%)	3 (33%)	1 (11%)	68 (28%)
**Professional caregiver**, ***n*** **(%)**							
Professional caregiver	27 (36%)	14 (26%)	7 (30%)	2 (22%)	2 (33%)	6 (75%)	58 (24%)
Missing	21 (22%)	35 (40%)	5 (18%)	3 (25%)	3 (33%)	1 (11%)	68 (28%)
**Hours of care required (total per week)**							
Mean (SD)	61.2 (49.5)	55.9 (57.7)	87.2 (106.2)	47.6 (34.8)	43.5 (32.7)	75.3 (50.9)	62.4 (61.7)
Missing	21 (22%)	35 (40%)	5 (18%)	3 (25%)	3 (33%)	1 (11%)	68 (28%)
Common types of assistance required from caregiver^*^							
Walking	83 (86%)	73 (83%)	20 (71%)	6 (55%)	9 (100%)	6 (67%)	197 (81%)
Getting washed and dressed	60 (63%)	34 (39%)	14 (50%)	3 (27%)	4 (44%)	7 (78%)	122 (50%)
Preparing meals/cooking	50 (57%)	40 (45%)	14 (50%)	2 (18%)	3 (33%)	6 (67%)	120 (50%)
Traveling out of home	53 (55%)	37 (42%)	15 (54%)	5 (45%)	4 (44%)	5 (56%)	119 (49%)
Help with shopping	52 (54%)	34 (39%)	17 (61%)	4 (36%)	4 (44%)	5 (56%)	116 (48%)
Emotional support	53 (55%)	34 (39%)	16 (57%)	5 (45%)	3 (33%)	4 (44%)	115 (48%)
Getting in and out of bed	54 (56%)	31 (35%)	12 (43%)	2 (18%)	2 (22%)	5 (56%)	106 (44%)
Organizing daily activities	46 (48%)	32 (36%)	12 (43%)	5 (45%)	4 (44%)	5 (56%)	104 (43%)
Motivation for daily activities	42 (44%)	33 (38%)	15 (54%)	4 (36%)	3 (33%)	6 (67%)	103 (43%)
Help with going to the toilet	48 (50%)	29 (33%)	9 (32%)	2 (18%)	5 (56%)	7 (78%)	100 (41%)

* *The 10 most frequently reported, overall, types of assistance are included*.

† *For four of the outcomes reported in this table data were missing for some patients*.

*Where relevant, the number of patients with missing data are reported below the relevant outcome. Percentages reported are based on the percentage of patients with data available. PSP, progressive supranuclear palsy; PSP-CBS, PSP-predominant corticobasal syndrome; PSP-F, PSP-predominant frontal presentation; PSP-P, PSP-predominant Parkinsonism; PSP-PGF, PSP-progressive gait freezing; PSP-RS, PSP-Richardson's syndrome; PSP-SL, PSP-predominant speech/language disorder; SD, standard deviation*.

Caregiver assistance was required with a wide range of daily tasks across all phenotypes, with the majority of patients (81%) requiring assistance with walking. At least 41% (range: 41–81%) of patients required assistance with carrying out a wide range of fundamental daily tasks, for all phenotypes.

### Healthcare System Burden

During a median time of 19 months since diagnosis, 75 patients (38%) reported hospitalizations due to PSP; among these patients the average number of hospitalizations was 2.3 (Table 4). The most common reasons for hospitalization were reported as falls (44% of patients), pneumonia or other infections (33%), and worsening of symptoms (24%). With few exceptions, patients of all phenotypes needed multiple HCPs to be involved in their management, covering a range of 12 different HCP types (list available in Table 4). In total, a mean of 3.6–4.4 HCP specializations, across phenotypes, was involved in the patient management. HCP utilization across countries was largely similar, with movement disorder specialists, primary care physicians, neurologists, and physical therapists comprising the most frequently involved specializations (Supplementary Table 1).

**Table 4:** Burden on healthcare system, by PSP phenotype.

	**PSP-RS**	**PSP-P**	**PSP-CBS**	**PSP-SL**	**PSP-PGF**	**PSP-F**	Total^*^
	**(*n* = 96)**	**(*n* = 88)**	**(*n* = 28)**	**(*n* = 12)**	**(*n* = 9)**	**(*n* = 9)**	**(*n* = 242)**
**Hospitalization history (since diagnosis)**							
Hospitalized due to PSP, *n* (%)	35 (43%)	20 (30%)	8 (36%)	5 (45%)	4 (44%)	3 (60%)	75 (38%)
Total number of hospitalizations, mean (SD)^†^	2.7 (2.5)	2.0 (1.3)	1.0 (0.0)	4.2 (3.8)	1.3 (0.5)	1.3 (0.6)	2.3 (2.2)
Missing, *n* (%)	14 (15%)	21 (24%)	6 (21%)	1 (8%)	0 (0%)	4 (44%)	46 (19%)
**HCP specializations involved in patient management**, ***n*** **(%)**							
Movement disorder specialist	68 (71%)	74 (84%)	24 (86%)	10 (83%)	8 (89%)	8 (89%)	192 (79%)
Primary care physician	69 (72%)	56 (64%)	17 (61%)	6 (50%)	6 (67%)	5 (56%)	159 (66%)
Neurologist	62 (65%)	39 (44%)	10 (36%)	6 (50%)	5 (56%)	6 (67%)	128 (53%)
Physical therapist	61 (64%)	36 (41%)	11 (39%)	3 (25%)	6 (67%)	4 (44%)	121 (50%)
Speech-language pathologist	40 (42%)	19 (22%)	7 (25%)	7 (58%)	3 (33%)	2 (22%)	78 (32%)
Social worker	18 (19%)	13 (15%)	8 (29%)	1 (8%)	2 (22%)	3 (33%)	45 (19%)
Occupational therapist	15 (16%)	17 (19%)	4 (14%)	2 (17%)	1 (11%)	4 (44%)	43 (18%)
Ophthalmologist	23 (24%)	11 (13%)	2 (7%)	2 (17%)	1 (11%)	0 (0%)	39 (16%)
Neurology nurse	13 (14%)	9 (10%)	3 (11%)	0 (0%)	1 (11%)	2 (22%)	28 (12%)
Psychologist	6 (6%)	10 (11%)	3 (11%)	4 (33%)	1 (11%)	1 (11%)	25 (10%)
Dietician	10 (10%)	7 (7%)	2 (7%)	2 (17%)	2 (22%)	1 (11%)	23 (10%)
Psychiatrist	3 (3%)	4 (5%)	3 (11%)	0 (0%)	0 (0%)	2 (22%)	12 (5%)
**Total number of HCP types involved in management**							
Mean (SD)	4.3 (2.0)	3.6 (1.9)	3.6 (2.2)	3.8 (2.5)	4.1 (2.1)	4.4 (2.7)	4.0 (2.1)
**Total HCP consultations (last 12 months)**							
Mean (SD)	11.6 (17.6)	13.3 (22.3)	6.9 (8.8)	6.8 (9.1)	12.9 (10.9)	6.0 (7.3)	11.3 (18.0)
**Provision of patient aids**, ***n*** **(%)**							
Mobility and/or visual aid	82 (86%)	70 (80%)	23 (82%)	6 (55%)	8 (89%)	8 (100%)	197 (82%)
Missing, *n* (%)	1 (1%)	0 (0%)	0 (0%)	1 (8%)	0 (0%)	1 (11%)	3 (1%)
**Other resource utilization**, ***n*** **(%)**							
Performed home modification	59 (73%)	56 (71%)	18 (67%)	6 (55%)	7 (78%)	5 (71%)	151 (71%)
Missing, *n* (%)	15 (16%)	9 (10%)	1 (4%)	1 (8%)	0 (0%)	2 (22%)	28 (12%)

* *For three of the outcomes reported in this table data were missing for some patients. Where relevant, the number of patients with missing data are reported below the relevant outcome. Percentages reported are based on the percentage of patients with data available*.

† *Percentage calculated from the subset of 75 patients who reported hospitalizations. HCP, healthcare professional/s; PSP, progressive supranuclear palsy; PSP-CBS, PSP-predominant corticobasal syndrome; PSP-F, PSP-predominant frontal presentation; PSP-P, PSP-predominant Parkinsonism; PSP-PGF, PSP-progressive gait freezing; PSP-RS, PSP-Richardson's syndrome; PSP-SL, PSP-predominant speech/language disorder; SD, standard deviation*.

To improve the quality of life of people living with PSP, the healthcare system provided mobility and/or visual aids to the majority (82%) of patients (55–100%, across phenotypes). The majority of patients (71%) also required a modification to their home to facilitate their needs (55–78%, across phenotypes). The rooms commonly adapted were the bathroom, bedroom, living room, and kitchen; installations of grab bars or railings, removal/movement of low objects, installation of ramps, and fitting of stairlifts were the most common adaptations.

## Discussion

Our descriptive analysis of contemporary data on 242 people living with PSP documents the high burden of PSP on patients, caregivers, and healthcare systems across the phenotypes studied. Few studies have reported data across multiple phenotypes, and these are usually restricted to comparisons between PSP-RS and PSP-P (2, 10, 18–20). Our data are the first to systematically report disease burden on the majority (6 out of 8) of the phenotypic variants defined according to the MDS criteria. There is no rational explanation why physicians would be less likely to report the two phenotypic variants not recorded in this study (PSP-PI, PSP-OM), since previous smaller-sized studies have identified/reported people living with these phenotypes (5). With regard to the phenotypes reported in this study, the distribution is similar to that reported by previous studies, in which PSP-RS was by far the most prevalent phenotype (5, 21).

In relation to patient burden, the majority of patients in our study had moderate-to-severe disease (67–100% by phenotype). Previous findings have consistently reported severe disability in comparable cohorts, for example, dell'Aquila et al. (2), evaluated people living with PSP-RS and PSP-P and reported 77% of patients had severe disability. However, no other studies have systematically reported patient burden for the wide spectrum of phenotypic variants of PSP. From the wide range of symptoms commonly observed in people living with PSP, this study confirms existing knowledge that mobility problems are the most common symptom (75–100% across phenotypes), and the motor domain appears to be the function most commonly impaired overall, across the phenotypes studied (76%). This is in line with previous evidence supportive of motor symptoms eventually affecting almost every person living with PSP (22, 23). The debilitating nature of the disease to people living with PSP was further illustrated in this study by 14 different symptom types being reported in >50% of all patients, as well as specific symptoms being reported in almost all patients, across all phenotypes (in particular difficulty walking, blepharospasm/involuntary blinking, rigidity, and dysphagia).

This evidence, however, must be considered while acknowledging the potential differences in pathogenesis and mechanisms that may lie behind the resulting symptom presentation (24, 25) as reported by the physicians for the different phenotypic variants. While all patients living with PSP in our cohort, regardless of phenotype, may present with certain symptoms (e.g., motor symptoms), these symptoms may have a differential evolution and severity of presentation/clinical course. It was recently highlighted, in a study using the Frontal Assessment Battery (FAB) neuropsychological examination, that the frontal lobe may act as a possible factor differentiating the PSP-P and PSP-RS variants as significant differences between the phenotypes were observed within the superior frontal gyrus medial of the dominant hemisphere (26). Comparable findings have been also reported in another recent study using FAB (27).

In terms of caregiver burden, our results on the proportion of patients requiring caregiving (62–100%, across phenotypes) is similar to previous results on the high overall level of caring required, as well as the majority of caregiving being provided informally at home (28). A recent study, which reported results on caregiver burden between PSP-RS and PSP-P, estimated *via* a caregiver burden scale, showed a very similar caregiver burden between phenotypes (20). The intense (in terms of hours of care per week) and comprehensive nature of assistance that our results suggest is required, covering all facets of daily function, is unsurprising considering the debilitating nature of all the phenotypic variants of PSP. These results also likely explain the high level of psychological burden, often leading to depression, documented in caregivers of people living with PSP (29). Considering that the most common comorbidity reported in people living with PSP in this study was also depression, it is feasible that in some scenarios both the patient and the caregiver experience depression. This is a critical issue considering the knowledge that poor mental health among caregivers is predictive of mortality of people living with neurodegenerative disease in general (30), but also specifically in PSP (31).

With regard to the burden on the healthcare system, our results highlight that multiple HCP specializations are required for patient care and management, incurring a substantial burden on healthcare systems, and that this is generally observed across all PSP phenotypes. This is a likely reflection of the lack of any effective pharmacological or non-pharmacological therapies available for PSP (32), translating to a lack of variation in treatment provided by phenotype. Finally, in relation to patient hospitalization history post-diagnosis, it is difficult to evaluate the observed potential differences between phenotypes (30–60% hospitalized, across phenotypes) considering the small numbers of patients hospitalized and the lack of studies systematically reporting hospitalization rates of people living with PSP on which these results could be benchmarked.

Our study's results suggest that movement disorder specialists are more likely to be involved in the management of patients with PSP compared with primary care physicians (79 vs. 66%). This finding confirms the recent Consensus Statement of the CurePSP Centers of Care (33) that suggests movement disorder specialists, within the multidisciplinary care team, assume the overall patient care. Our results also highlight the potential underutilization of HCPs specializing in speech and language pathology. Only 32% of patients with PSP had speech-language pathologists involved in their care despite 79% of patients with PSP presenting with dysphagia; since our study confirms previously reported presentation frequencies of dysphagia (34), we believe this finding is not due to an overestimate of the symptom presentation. Our finding further emphasizes the importance of recent recommendations of early assessment by speech and language therapy professionals (35).

Overall, our study's findings on the burden of different PSP phenotypes must be considered alongside the existing findings of the clinical manifestations of these phenotypes. Our findings align with the expectations derived from the clinical literature; for example, as was expected, our results report less frequent cognitive symptom burden for patients with PSP-PGF, whom cognition is known to be less impacted compared with other phenotypes (27, 35). On the other hand, as was recently reported in a study focusing on the cognitive and behavioral profile of PSP and its phenotypes (27), no major differences are observed between phenotypes in terms of behavioral/neuropsychological burden. Furthermore, although patients with PSP-SL were provided with care from a speech-language pathologist at almost twice the frequency of other phenotypes, our study reports that they are less frequently provided with care for getting washed and dressed, getting in and out of bed, help with going to the toilet or preparing meals/cooking; this finding could be explained by the fact that these patients may only later develop the PSP-typical motor features (36). Finally, our findings relating to patient age at diagnosis are also in line with previous findings that report that patients with PSP-PGF are significantly older than patients of other phenotypes (27).

One key strength of our study is that we captured detailed data in a consistent manner on a large number of people living with PSP from routine clinical settings, in six countries, using validated approaches. Moreover, we report clinical characteristics and burden to patients, caregivers, and healthcare systems across multiple PSP phenotypes for the first time.

There are limitations to this study that warrant further consideration. When using the DSP™ approach, the patient sample collected is not a random sample of patients, since the next “n” consulting patients are included. As such, they may not be fully representative of the overall patient population, as patients who consult frequently are more likely to be included. Also, although physicians are requested to collect data on a series of consecutive patients to avoid selection bias, this is contingent upon the integrity of the participating physician rather than formalized source verification procedures. Furthermore, while minimal inclusion criteria influenced the selection of neurologists/movement disorder specialists (mainly focusing on ensuring they consult a minimum number of people living with PSP and be actively involved in their management and treatment decisions), physician inclusion is, nevertheless, influenced by willingness to participate. Hence, physicians surveyed represent a pragmatic sample and are not representative of the overall population of physicians treating PSP.

The quality of our data depends primarily on the accurate reporting of information by physicians. Diagnosis in the target patient group is based primarily on the judgement and diagnostic skills of the respondent physician and a formalized diagnostic checklist is not mandated as part of the DSP™ methodology. However, this is entirely consistent with the diagnostic decisions made by physicians in routine clinical practice and is therefore reflective of the “real world". The same limitation may apply to the clinical judgement of patient disease severity by consulting movement disorder specialists/neurologists in our study. Although the reliability of the clinical judgement was not formally assessed, physician experience (>75% of specialists in our study have >15 years of medical practice; data based on physician self-report in the study questionnaire) and recent patient engagement (the majority of physicians consulted with the patient and/or their caregiver on the same day they completed the study questionnaire) reduce the likelihood of an erroneous assessment by a physician.

In terms of patient medical history, the DSP™ does not request all available patient medical records, which may result in the missingness of some medical information for the patient. However, this limitation is mitigated to a certain extent by the fact that the physician-completed PRFs are very detailed. Furthermore, while only 27% of eligible people living with PSP had a reported PSP phenotype, demographic characteristics were very similar to those without a reported PSP phenotype (70.2 vs. 68.4 years mean age; 58 vs. 62% males, respectively) suggesting that the study patient sample is representative of the wider patient population. One potential explanation may relate to lack of an effective treatment available for PSP, limiting the perceived utility to certain physicians of distinguishing/ascertaining the specific PSP phenotype of patients.

Furthermore, since the DSP™ approach is a point-in-time survey providing a snapshot post PSP diagnosis, patients were characterized at different times since their diagnosis (median range 8.0–22.5 months, across phenotypes), and so patients may have been at different stages of their disease progression. This issue may confound the results reported by phenotype. In this study, however, disease severity was reported by physicians as being primarily moderate or severe across all phenotypes, suggesting that overall, few patients may have been at the very early stages of disease at the time of data collection. One potential explanation for this finding is that disease severity, across phenotypes, presents/progresses to moderate or severe within the first 1–2 years (2, 37). In general, the existing scientific literature acknowledges that across phenotypes, multiple severely debilitating symptoms may appear early in the course of the disease (38).

To be included in the study, patients had to have a confirmed diagnosis of PSP by their respective neurologist/movement disorder specialist only. It was not possible to perform a post-mortem histopathological confirmation of cases, which would have provided the optimal level of diagnostic certainty. However, since our study only includes patients with a physician-confirmed PSP phenotypic variant, we expect that these are patients that have been diagnosed by neurologists/movement disorder specialists who are informed about PSP phenotypic variants and the respective MDS criteria. In this respect, it must be acknowledged that specific PSP subtypes may represent misdiagnoses due to similarities in their clinical presentation with other neurodegenerative diseases, such as Multiple System Atrophy (MSA), Parkinson's disease, and Dementia with Lewy bodies (39, 40). For example, early diagnostic discrimination between PSP-P and the parkinsonian subtype of MSA is particularly challenging (9, 41), an issue which may have influenced our results in relation to the PSP-P subtype in this study.

Our findings may also have been influenced by country-specific factors, since healthcare practices are known to differ between countries. Although geographical differences in burden were not one of the study objectives and hence not evaluated, the inclusion of six countries from Western Europe/North America where people living with PSP are known to receive comparable treatments/healthcare procedures, means we expect such factors not to have largely influenced our results. This is further supported by the fact that similar specialist HCPs were utilized across the countries (based on *post-hoc* analyses; Supplementary Table 1).

In conclusion, our study highlights the high burden on patients, caregivers, and healthcare systems across all PSP phenotypes. Although phenotypes are known to manifest different symptoms and are associated with different diagnostic pathways, all people living with PSP are observed to typically receive similar care and impact healthcare systems in similar ways. These results suggest that the urgent unmet need for disease-modifying agents, more effective symptomatic treatments, and improved patient management strategies applies to all phenotypic variants of PSP. Future studies should aim to build on our findings by collecting longitudinal data across all phenotypic variants to investigate how patient burden may differentiate over time by variant.

## Data Availability Statement

The data analyzed in this study were obtained from Adelphi Real World, the following licenses/restrictions apply: Requests to access these datasets must first be granted by Adelphi Real World. Requests to access these datasets should be directed to JM, jennifer.mellor@adelphigroup.com.

## Ethics Statement

The studies involving human participants were reviewed and approved by Salus Institutional Review Board, study protocol number AG 8445. The patients/participants provided their written informed consent to participate in this study.

## Author Contributions

DP, AK, TG, AA, AT, JW, JM, and AS designed the study. DP, JW, JM, and AS were responsible for its organization and execution. DP, JW, JM, and AS designed the statistical analysis, which was performed by JW and JM and reviewed by DP, AK, TG, AA, AT, and AS. DP and AS prepared the initial draft of the manuscript, which was reviewed and critiqued by all authors. All authors contributed to the article and approved the submitted version.

## Funding

This study was funded by UCB Pharma.

## Conflict of Interest

DP, AK, TG, AA, AT, and AS were employed by UCB Pharma during the course of this work and may hold/have access to stock options. JW and JM are employees of Adelphi Real World.

## Publisher's Note

All claims expressed in this article are solely those of the authors and do not necessarily represent those of their affiliated organizations, or those of the publisher, the editors and the reviewers. Any product that may be evaluated in this article, or claim that may be made by its manufacturer, is not guaranteed or endorsed by the publisher.

## References

[1] National Institute of Neurological Disorders Stroke (NINDS). Progressive Supranuclear Palsy Fact Sheet. (2020). Available online at: https://www.ninds.nih.gov/Disorders/Patient-Caregiver-Education/Fact-Sheets/Progressive-Supranuclear-Palsy-Fact-Sheet (accessed June 15, 2022).

[2] dell'Aquila C Zoccolella S Cardinali V De Mari M Iliceto G Tartaglione B. Predictors of survival in a series of clinically diagnosed progressive supranuclear palsy patients. Parkinsonism Relat Disord. (2013) 19:980–5. 10.1016/j.parkreldis.2013.06.014 23968651

[3] Williams DR Litvan I. Parkinsonian syndromes. Continuum. (2013) 19:1189–212. 10.1212/01.CON.0000436152.24038.e0 24092286 PMC4234134

[4] Steele JC Richardson JC Olszewski J. Progressive supranuclear palsy. A heterogeneous degeneration involving the brain stem, basal ganglia and cerebellum with vertical gaze and pseudobulbar palsy, nuchal dystonia and dementia. Arch Neurol. (1964) 10:333–59. 10.1001/archneur.1964.00460160003001 14107684

[5] Respondek G Stamelou M Kurz C Ferguson LW Rajput A Chiu WZ. The phenotypic spectrum of progressive supranuclear palsy: a retrospective multicenter study of 100 definite cases. Mov Disord. (2014) 29:1758–66. 10.1002/mds.26054 25370486

[6] Hoglinger GU Respondek G Stamelou M Kurz C Josephs KA Lang AE. Clinical diagnosis of progressive supranuclear palsy: the movement disorder society criteria. Mov Disord. (2017) 32:853–64. 10.1002/mds.26987 28467028 PMC5516529

[7] Boxer AL Yu JT Golbe LI Litvan I Lang AE Hoglinger GU. Advances in progressive supranuclear palsy: new diagnostic criteria, biomarkers, and therapeutic approaches. Lancet Neurol. (2017) 16:552–63. 10.1016/S1474-4422(17)30157-6 28653647 PMC5802400

[8] Chen S Jakab I Zelei T Szilberhorn L Bendes R Elezbawy B. Burden of progressive supranuclear palsy: a systematic literature review. Mov Disord. (2018) 33 (suppl 2):S414–5. abstract 921.28315409

[9] Alster P Madetko N Koziorowski D Friedman A. Progressive supranuclear palsy—parkinsonism predominant (PSP-P)—a clinical challenge at the boundaries of psp and Parkinson's disease (PD). Front Neurol. (2020) 11:180. 10.3389/fneur.2020.00180 32218768 PMC7078665

[10] Williams DR De Silva R Paviour DC Pittman A Watt HC Kilford L. Characteristics of two distinct clinical phenotypes in pathologically proven progressive supranuclear palsy: Richardson's syndrome and PSP-parkinsonism. Brain. (2005) 128:1247–58. 10.1093/brain/awh488 15788542

[11] Caso F Agosta F Ječmenica-Lukić M Petrović I Meani A Kostic VS. Progression of white matter damage in progressive supranuclear palsy with predominant parkinsonism. Parkinsonism Relat Disord. (2018) 49:95–9. 10.1016/j.parkreldis.2018.01.001 29336906

[12] Mahale RR Krishnan S Divya KP Jisha VT Kishore A. Subtypes of PSP and prognosis: a retrospective analysis. Ann Indian Acad Neurol. (2021) 24:56–62. 10.4103/aian.AIAN_611_20 33911380 PMC8061531

[13] Anderson P Benford M Harris N Karavali M Piercy J. Real-world physician and patient behavior across countries: disease-specific programmes - a means to understand. Curr Med Res Opin. (2008) 24:3063–72. 10.1185/03007990802457040 18826746

[14] Morgan JC Ye X Mellor JA Golden KJ Zamudio J Chiodo LA. Disease course and treatment patterns in progressive supranuclear palsy: a real-world study. J Neurol Sci. (2021) 421:117293. 10.1016/j.jns.2020.117293 33385754

[15] US Department of Health and Human Services. Summary of the HIPAA Privacy Rule. (2003). Available online at: http://www.hhs.gov/sites/default/files/privacysummary.pdf (accessed January 5, 2021).

[16] Health Information Technology. Health Information Technology Act. (2009). Available online at: https://www.healthit.gov/sites/default/files/hitech_act_excerpt_from_arra_with_index.pdf (accessed January 5, 2021).

[17] European Pharmaceutical Market Research Association (EphMRA). (2019). Available online at: https://www.ephmra.org/standards/code-of-conduct/ (accessed January 5, 2021).

[18] Jecmenica-Lukic M Petrovic IN Pekmezovic T Kostic VS. Clinical outcomes of two main variants of progressive supranuclear palsy and multiple system atrophy: a prospective natural history study. J Neurol. (2014) 261:1575–83. 10.1007/s00415-014-7384-x 24888315

[19] Shoeibi A Litvan I Juncos JL Bordelon Y Riley D Standaert D. Are the international parkinson disease and movement disorder society progressive supranuclear palsy (IPMDS-PSP) diagnostic criteria accurate enough to differentiate common PSP phenotypes? Parkinsonism Relat Disord. (2019) 69:34–9. 10.1016/j.parkreldis.2019.10.012 31665686 PMC6914266

[20] Chaithra SP Prasad S Holla VV Stezin A Kamble N Yadav R. The Non-motor symptom profile of progressive supranuclear palsy. J Mov Disord. (2020) 13:118–26. 10.14802/jmd.19066 32241079 PMC7280946

[21] Jabbari E Holland N Chelban V Jones PS Lamb R Rawlinson C. Diagnosis across the spectrum of progressive supranuclear palsy and corticobasal syndrome. JAMA Neurol. (2020) 77:377–87. 10.1001/jamaneurol.2019.4347 31860007 PMC6990759

[22] Santacruz P Uttl B Litvan I Grafman J. Progressive supranuclear palsy: a survey of the disease course. Neurology. (1998) 50:1637–47. 10.1212/WNL.50.6.1637 9633705

[23] Arena JE Weigand SD Whitwell JL Hassan A Eggers SD Hoglinger GU. Progressive supranuclear palsy: progression and survival. J Neurol. (2016) 263:380–9. 10.1007/s00415-015-7990-2 26705121

[24] Kovacs GG Lukic MJ Irwin DJ Arzberger T Respondek G Lee EB. Distribution patterns of tau pathology in progressive supranuclear palsy. Acta Neuropathol. (2020) 140:99–119. 10.1007/s00401-020-02158-2 32383020 PMC7360645

[25] Whitwell JL Tosakulwong N Botha H Ali F Clark HM Duffy JR. Brain volume and flortaucipir analysis of progressive supranuclear palsy clinical variants. Neuroimage Clin. (2020) 25:102152. 10.1016/j.nicl.2019.102152 31935638 PMC6961761

[26] Alster P Migda B Madetko N Duszyńska-Was K Drzewińska A Charzyńska I. The role of frontal assessment battery and frontal lobe single-photon emission computed tomography in the differential diagnosis of progressive supranuclear palsy variants and corticobasal syndrome-a pilot study. Front Neurol. (2021) 12:630153. 10.3389/fneur.2021.630153 33613435 PMC7891101

[27] Horta-Barba A Pagonabarraga J Martínez-Horta S Busteed L Pascual-Sedano B Illán-Gala I. Cognitive and behavioral profile of progressive supranuclear palsy and its phenotypes. J Neurol. (2021) 268:3400–8. 10.1007/s00415-021-10511-y 33704556

[28] Hensler M Paul S Abright C Lorenzl S. Progressive supranuclear palsy: living environment of the patients in Germany. Nervenarzt. (2011) 82:207–14. 10.1007/s00115-010-3076-7 20669002

[29] Schmotz C Richinger C Lorenzl S. High burden and depression among late-stage idiopathic parkinson disease and progressive supranuclear palsy caregivers. J Geriatr Psychiatry Neurol. (2017) 30:267–72. 10.1177/0891988717720300 28747135

[30] Lwi SJ Ford BQ Casey JJ Miller BL Levenson RW. Poor caregiver mental health predicts mortality of patients with neurodegenerative disease. Proc Natl Acad Sci USA. (2017) 114:7319–24. 10.1073/pnas.1701597114 28655841 PMC5514722

[31] Shea YF Shum ACK Lee SC Chiu PKC Leung KS Kwan YK. Natural clinical course of progressive supranuclear palsy in Chinese patients in Hong Kong. Hong Kong Med J. (2019) 25:444–52. 10.12809/hkmj198101 31796642

[32] Hajjar SH Cooper JK. Progressive supranuclear palsy treatment - a systematic review. Basal Ganglia. (2016) 6:75–8. 10.1016/j.baga.2016.01.004

[33] Bluett B Pantelyat AY Litvan I Ali F Apetauerova D Bega D. Best practices in the clinical management of progressive supranuclear palsy and corticobasal syndrome: a consensus statement of the CurePSP centers of care. Front Neurol. (2021) 12:694872. 10.3389/fneur.2021.694872 34276544 PMC8284317

[34] Clark HM Stierwalt JAG Tosakulwong N Botha H Ali F Whitwell JL. Dysphagia in progressive supranuclear palsy. Dysphagia. (2020) 35:667–76. 10.1007/s00455-019-10073-2 31676923 PMC7192765

[35] Rowe JB Holland N Rittman T. Progressive supranuclear palsy: diagnosis and management. Pract Neurol. (2021) 21:376–83. 10.1136/practneurol-2020-002794 34215700 PMC8461411

[36] Peterson KA Patterson K Rowe JB. Language impairment in progressive supranuclear palsy and corticobasal syndrome. J Neurol. (2021) 268:796–809. 10.1007/s00415-019-09463-1 31321513 PMC7914167

[37] Payan CA Viallet F Landwehrmeyer BG Bonnet AM Borg M Durif F. Disease severity and progression in progressive supranuclear palsy and multiple system atrophy: validation of the NNIPPS–Parkinson plus scale. PLoS ONE. (2011) 6:e22293. 10.1371/journal.pone.0022293 21829612 PMC3150329

[38] Respondek G Hoglinger GU. The phenotypic spectrum of progressive supranuclear palsy. Parkinsonism Relat Disord. (2016) 22 Suppl 1:S34–6. 10.1016/j.parkreldis.2015.09.041 26421392

[39] Koga S Aoki N Uitti RJ Van Gerpen JA Cheshire WP Josephs KA. When DLB, PD, and PSP masquerade as MSA: an autopsy study of 134 patients. Neurology. (2015) 85:404–12. 10.1212/WNL.0000000000001807 26138942 PMC4534078

[40] Poewe W Seppi K Tanner CM Halliday GM Brundin P Volkmann J. Parkinson disease. Nat Rev Dis Primers. (2017) 3:17013. 10.1038/nrdp.2017.13 28332488

[41] Alster P Nieciecki M Migda B Kutyłowski M Madetko N Duszyńska-Was K. The strengths and obstacles in the differential diagnosis of progressive supranuclear palsy-Parkinsonism predominant (PSP-P) and multiple system atrophy (MSA) using magnetic resonance imaging (MRI) and perfusion single photon emission computed tomography (SPECT). Diagnostics. (2022) 12:385. 10.3390/diagnostics12020385 35204476 PMC8871165

