# Supplementary material for: The Burden of Progressive Supranuclear Palsy on Patients, Caregivers, and Healthcare Systems by PSP Phenotype: A Cross-Sectional Study
Source: Front Neurol. 2022 Jul 4;13:821570. doi: 10.3389/fneur.2022.821570 (PMC9295700; doi:10.3389/fneur.2022.821570)
Supplement: Supplementary file 1 [file Table_1.DOCX]

**Supplementary Table 1 |** HCP specializations involved in patient management across different countries

|  | **USA**  **(*n* = 37)** | **France**  **(*n* = 30)** | **Germany**  **(*n* = 48)** | **Italy**  **(*n* = 48)** | **Spain  (n = 36)** | **UK (n = 43)** | **EU5**  **(*n* = 205)** | **Total**  **(*n* = 242)** |
| --- | --- | --- | --- | --- | --- | --- | --- | --- |
| **HCP specializations involved in patient management, *n* (%)** | | | | | | | | |
| Movement disorder specialist | 27 (73) | 24 (80) | 37 (77) | 40 (83) | 31 (86) | 33 (77) | 165 (80) | 192 (79) |
| Primary care physician | 15 (41) | 22 (73) | 44 (92) | 30 (63) | 21 (58) | 27 (63) | 144 (70) | 159 (66) |
| Neurologist | 23 (62) | 15 (50) | 26 (54) | 25 (52) | 17 (47) | 22 (51) | 105 (51) | 128 (53) |
| Neuropsychiatrist | 3 (8) | 0 (0) | 1 (2) | 1 (2) | 0 (0) | 3 (7) | 5 (2) | 8 (3) |
| Physical therapist | 21 (57) | 18 (60) | 34 (71) | 27 (56) | 7 (19) | 14 (33) | 100 (49) | 121 (50) |
| Speech-language pathologist | 11 (30) | 13 (43) | 23 (48) | 14 (29) | 0 (0) | 17 (40) | 67 (33) | 78 (32) |
| Social worker | 4 (11) | 8 (27) | 7 (15) | 5 (10) | 10 (28) | 11 (26) | 41 (20) | 45 (19) |
| Occupational therapist | 13 (35) | 3 (10) | 12 (25) | 2 (4) | 1 (3) | 12 (28) | 30 (15) | 43 (18) |
| Ophthalmologist | 9 (24) | 1 (3) | 15 (31) | 6 (13) | 7 (19) | 1 (2) | 30 (15) | 39 (16) |
| Neurology nurse | 1 (3) | 2 (7) | 4 (8) | 6 (13) | 2 (6) | 13 (30) | 27 (13) | 28 (12) |
| Psychologist | 1 (3) | 7 (23) | 0 (0) | 9 (19) | 2 (6) | 6 (14) | 24 (12) | 25 (10) |
| Dietician | 1 (3) | 3 (10) | 2 (4) | 8 (17) | 1 (3) | 8 (19) | 22 (11) | 23 (10) |
| Psychiatrist | 1 (3) | 0 (0) | 3 (6) | 3 (6) | 3 (8) | 2 (5) | 11 (5) | 12 (5) |
| Other | 0 (0) | 2 (7) | 0 (0) | 2 (4) | 3 (8) | 6 (14) | 13 (6) | 13 (5) |
| **Total number of HCP types involved in management** | | | | | | | | |
| Mean (SD) | 3.8 (2.2) | 4.0 (1.7) | 4.5 (1.7) | 3.9 (2.2) | 3.1 (1.6) | 4.3 (2.6) | 4 (2.1) | 4.0 (2.1) |

*HCP, healthcare professional/s; EU5, France, Germany, Italy, Spain and the United Kingdom; SD, standard deviation; USA, United States of America; UK, United Kingdom.*
